# IBtkα Activates the β-Catenin-Dependent Transcription of *MYC* through Ubiquitylation and Proteasomal Degradation of GSK3β in Cancerous B Cells

**DOI:** 10.3390/ijms23042044

**Published:** 2022-02-12

**Authors:** Eleonora Vecchio, Nancy Nisticò, Gaetanina Golino, Enrico Iaccino, Domenico Maisano, Selena Mimmi, Annamaria Aloisio, Maurizio Renna, Angelica Avagliano, Alessandro Arcucci, Giuseppe Fiume, Ileana Quinto

**Affiliations:** 1 Department of Experimental and Clinical Medicine, University of Catanzaro ‘Magna Graecia’, 88100 Catanzaro, Italy; nancynistico@unicz.it (N.N.); tania.golino@gmail.com (G.G.); iaccino@unicz.it (E.I.); maisano@unicz.it (D.M.); mimmi@unicz.it (S.M.); aloisio@unicz.it (A.A.); quinto@unicz.it (I.Q.); 2 Department of Molecular Medicine and Medical Biotechnology, University of Naples Federico II, 80131 Naples, Italy; maurizio.renna@unina.it; 3 Department of Public Health, University of Naples Federico II, 80131 Naples, Italy; angelica.avagliano@unina.it (A.A.); alessandro.arcucci2@unina.it (A.A.)

**Keywords:** *MYC*, β-catenin, GSK3β, Burkitt’s lymphoma, IBtkα

## Abstract

The *IBTK* gene encodes the IBtkα protein that is a substrate receptor of E3 ubiquitin ligase, Cullin 3. We have previously reported the pro-tumorigenic activity of *Ibtk* in *MYC*-dependent B-lymphomagenesis observed in *Eμ-myc* transgenic mice. Here, we provide mechanistic evidence of the functional interplay between IBtkα and *MYC*. We show that IBtkα, albeit indirectly, activates the β-catenin-dependent transcription of the *MYC* gene. Of course, IBtkα associates with GSK3β and promotes its ubiquitylation, which is associated with proteasomal degradation. This event increases the protein level of β-catenin, a substrate of GSK3β, and results in the transcriptional activation of the *MYC* and *CCND1* target genes of β-catenin, which are involved in the control of cell division and apoptosis. In particular, we found that in Burkitt’s lymphoma cells, IBtkα silencing triggered the downregulation of both *MYC* mRNA and protein expression, as well as a strong decrease of cell survival, mainly through the induction of apoptotic events, as assessed by using flow cytometry-based cell cycle and apoptosis analysis. Collectively, our results shed further light on the complex puzzle of IBtkα interactome and highlight IBtkα as a potential novel therapeutic target to be employed in the strategy for personalized therapy of B cell lymphoma.

## 1. Introduction

The *Inhibitor of Bruton’s tyrosine kinase* (*IBTK*) gene encodes the protein isoforms IBtkα, IBtkβ, and IBtkγ [1,2]. The 26 kDa IBtkγ protein was originally characterized as an inhibitor of Bruton’s tyrosine kinase (Btk) [3], an essential enzyme required for BCR signalling. IBtkα is the most abundant and biologically relevant protein isoform and shares a high homology with the murine *Ibtk* [1]. We, and other research groups, have demonstrated that IBtkα is required for survival of different cell types [4,5,6]. In particular, the expression of IBtkα increased in the aggressive stage of B-chronic lymphocytic leukemia (CLL) cells, while it decreased the disease remission phase following to chemotherapy [6]. Recent emerging studies confirmed that IBtkα silencing had the potential to induce apoptosis in hematological malignancies. Indeed, IBtkα RNA interference increased the apoptosis of CLL cells in response to chemotherapeutic agents [6]. Furthermore, knockout of the *Ibtk* gene in *Eμ-myc*-transgenic mice delayed the onset of B-lymphomas as a consequence of increased B-cell apoptosis [7]. IBtkα affects multiple pathways, including protein turnover, being a substrate receptor of Cullin 3 Ubiquitin ligase complex (CRL3^IBTK^). It promoted the proteasomal degradation of Pdcd4, a translation repressor interacting with the eIF4A1 helicase [8]. Notably, IBtkα RNA interference modified the transcriptome profile of HeLa and K562 cells, likely as a consequence of the functional interaction of IBtkα with proteins involved in transcription regulation [9]. 

*MYC* is a transcription factor that activates the expression of several genes, most of them involved in cell cycle regulation, apoptosis, and cellular transformation. In hematological malignancies, genetic and epigenetic alterations of the *MYC* gene increase the tumorigenic potential of *MYC*, resulting in the transcriptional upregulation of *MYC* target genes [10,11]. Of interest for this study, *MYC* enhanced the expression of the *Ibtk* gene in B cells of *Eμ-myc* transgenic mice [7], and this was allowed by its direct binding to the *Ibtk* promoter, demonstrated by ChIP-seq analysis [12]. These findings suggested a functional cross-talk between *Ibtk* and *MYC* in a murine model of B-lymphomagenesis [13]. To date, the direct contribution of IBtkα to the growth of *MYC*-driven B lymphomas and the molecular mechanism leading to the synergistic action of IBtkα and *MYC* have never been reported. 

Several studies are ongoing to better understand the importance of the interaction of *MYC* with other oncogenes. For instance, it has been shown that the expression of *MYC* exerted the highest tumorigenic effect when the β-catenin pathway was activated. Indeed, the expression of the *MYC* gene is under the control of β-catenin and is negatively regulated through cytoplasmic retention and degradation operated by the β-catenin destruction complex, which includes the Ser/Thr kinases glycogen synthase kinase 3 β (GSK3 β) and casein kinase-1, the tumor suppressors Axin and adenomatous polyposis coli (APC), the protein phosphatase 2A, and the E3-ubiquitin ligase β-TrCP [14]. In particular, GSK3β phosphorylates β-catenin, tagging it for ubiquitination coupled to proteasomal degradation [14]. The secreted glycoproteins of the Wnt family activate the β-catenin activity as they bind to Frizzled receptors and LRP co-receptors, and promote the membrane recruitment of Disheveled proteins, which ultimately inhibit GSK3β [15]. As a consequence, β-catenin is released from the β-catenin destruction complex in the cytoplasm and translocates to the nucleus, where it interacts with T cell factor/lymphoid-enhancing factor (Tcf/Lef) transcription factors to promote the expression of target genes, including *MYC* [16,17,18]. In this context, the upregulation of the β-catenin signaling has been associated with tumorigenesis and metastasis [14,19], supporting its relevant role in the transcriptional control of tumorigenic functions. 

Differently from previous studies that have shown *IBTK* as a potential transcriptional target of *MYC* in *MYC*-driven B-cell lymphomas [7,12], in this study, we raised the hypothesis of an upstream effect exerted by IBtkα in regulating the expression of *MYC* gene. To this end, we analyzed the effects of IBtkα silencing on the development of *MYC*-driven B lymphoma in order to determine whether IBTKα-GSK3β interaction and β-catenin are involved in this process. In particular, we found that the IBtkα silencing triggered the downregulation of both *MYC* mRNA and protein expression, as well as a strong decrease of cell survival, mainly through the induction of apoptotic events in Burkitt’s lymphoma cells.

## 2. Results

### 2.1. IBtkα Silencing Reduces the Expression of MYC Gene in Burkitt’s Lymphoma Cells

**Figure 1:**
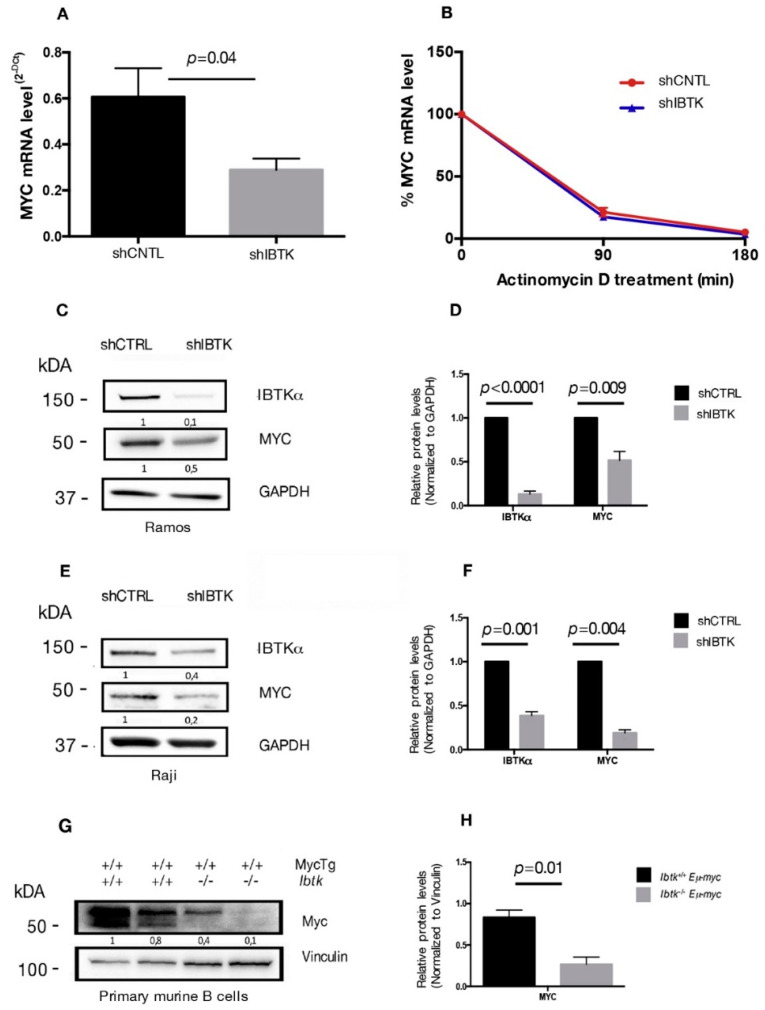
IBtkα silencing reduces the gene and protein expression of *MYC*. (**A**) Ramos cells (3 × 10^6^) were transduced with lentiviral particles (500 ng of p24) expressing shCNTL or shIBTK. Twenty-four hours later, infected Ramos cells were selected with puromycin (1.5 μg/mL) for 48 h. Total RNA was analyzed by qRT-PCR for the expression of *MYC* transcripts and normalized to GAPDH transcripts. Values (mean ± SE, *n* = 3) are shown. Statistically significant difference was calculated according to Student’s *t* test. (**B**) Ramos cells (3 × 10^6^) were transduced with shCNTL or shIBTK and treated with actinomycin D (1 µM) for the indicated time. Total RNA was analyzed by qRT-PCR for the expression of *MYC* transcripts and normalized to GAPDH transcripts. Values (mean ± SE, *n* = 3) are shown. No statistically significant difference was observed according Student’s *t* test. (**C**) Ramos cells (3 × 10^6^) were transduced with shCNTL or shIBTK, and 24 h post-transduction they were selected with puromycin (1.5 μg/mL) for 48 h. Whole cell extracts (30 µg) were separated by Nupage 4–12% polyacrylamide gel and analyzed by Western blotting using the anti-IBtkα, anti-Myc, and anti-GAPDH antibodies. (**D**) Densitometric values of *IBTK* and *MYC* bands were normalized to GAPDH bands. Values (mean ± SE, *n* = 3) are shown. Statistically significant difference was calculated according to Student’s *t* test. (**E**) Raji cells (3 × 10^6^) were transduced with shCNTL or shIBTK, and 24 h post-transduction they were selected with puromycin (0.2 μg/mL) for 48 h. Whole cell extracts (30 µg) were separated by Nupage 4–12% polyacrylamide gel and analyzed by Western blotting using the anti-IBtkα, anti-Myc, and anti-GAPDH antibodies. (**F**) Densitometric values of *IBTK* and *MYC* bands were normalized to GAPDH bands. Values (mean ± SE, *n* = 3) are shown. Statistically significant difference was calculated according to Student’s *t* test. (**G**) Murine B cells were isolated from tumor lymph nodes of n.2 *Ibtk^+/+^ Eμ-myc* and n.2 *Ibtk^−/−^ Eμ-myc* mice. Mouse genotypes +/+ indicate wildtypes, while mouse genotypes −/− indicate homozygous mutants. Whole cell extracts (30 µg) were separated by Nupage 4–12% polyacrylamide gel and analyzed by Western blotting using the anti-Myc and anti-vinculin antibodies. (**H**) Densitometric values of *MYC* protein bands were normalized to vinculin bands. Values (mean ± SE, *n = 2*) are shown. Statistically significant difference was determined by using the Student’s *t* test.

Previous studies have shown that IBtkα is a potential transcriptional target of *MYC* [7,12] and that IBtkα can modulate the genome-wide expression in different cellular contexts [9]. Therefore, we asked whether IBtkα could directly or indirectly be involved in the transcriptional regulation of *MYC*, enhancing the oncogenic activity of *MYC*. To this end, we analyzed the *MYC* mRNA expression in Ramos cells, a human Burkitt’s lymphoma cell line that showed a resistance to chemotherapeutic drugs [20], in the presence or absence of IBtkα. Cells were transduced with lentiviral particles expressing short hairpin RNA against IBTKα mRNA (shIBTK) or control short hairpin RNA (shCNTL). Through RT-PCR analysis performed on total RNA samples, we found that the *MYC* transcripts were reduced by 50% in IBtkα-silenced cells, as compared to control cells (Figure 1A). To determine whether the decreased *MYC* transcription in the absence of IBtkα was due to reduced mRNA stability, cells silenced or not for IBtkα were treated with actinomycin D, a nucleic acid synthesis inhibitor, followed by a time-course RT-PCR of total RNA. The decay of *MYC* mRNA levels were similar in presence or absence of IBtkα (Figure 1B), therefore ruling out a role of IBtkα in the transcriptional regulation of *MYC* expression.

As an additional experiment, total protein extracts of Ramos cells, silenced or not, for IBtkα were analyzed by Western blotting for the expression levels of *MYC* and IBtkα proteins. A significant decrease of *MYC* and IBtkα was observed in IBtkα-silenced cells as compared to control cells (Figure 1C,D). Consistent with these observations, *MYC* expression was reduced also in Raji cells, another human Burkitt’s lymphoma cell line, and in primary B-cell lymphoma extracts from *Ibtk^−/−^Eμ-myc* mice, compared to *Ibtk^+/+^Eμ-myc* tumorigenic mice (Figure 1E–H). Taken together, these results demonstrate that the depletion of IBtkα causes a strong reduction of *MYC* expression, suggesting that IBtkα is able to sustain *MYC* expression in cancerous B cells.

### 2.2. IBtkα/GSK3β Interaction Promotes GSK3β Ubiquitylation and Its Proteasomal Degradation

The aberrant activation of β-catenin signaling is related to different hematological malignancies and *MYC* is a well-known downstream target of β-catenin signaling [21]. β-catenin is a transcription factor that is negatively regulated by the β-catenin destruction complex [14]. As a component of the β-catenin destruction complex, GSK3β phosphorylates β-catenin and this post-translational modification tags β-catenin for proteasomal degradation [14]. Using mass spectrometry, we have previously identified GSK3β as an interactor of IBtkα (Table S1) [8]. Thus, we reasoned that GSK3β could be a target of ubiquitylation and proteasomal degradation mediated by IBtkα, affecting the GSK3β inhibition of β-catenin.

**Figure 2:**
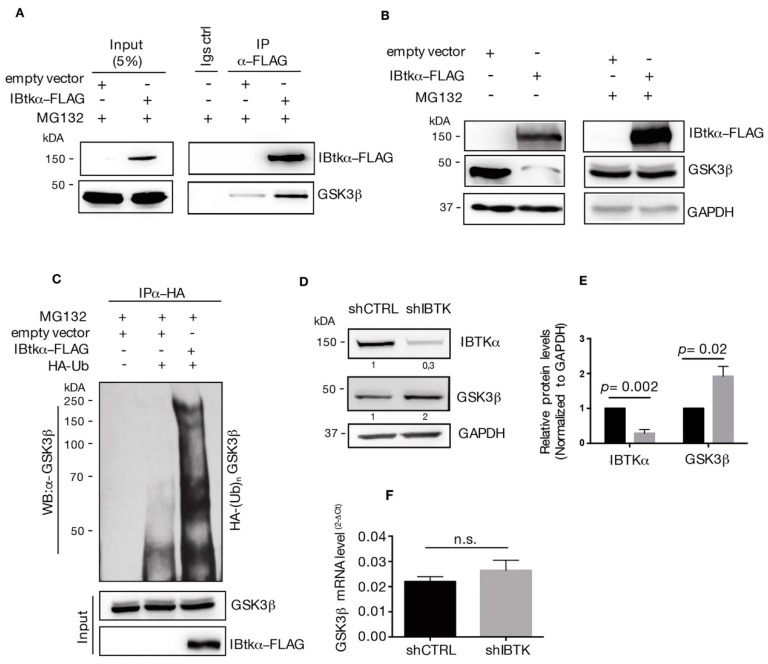
IBtkα interacts with GSK3β and promotes its poly-ubiquitylation and proteasomal degradation. (**A**) HEK293T cells (3 × 10^6^) were transfected with pCMV6-IBtkα-FLAG or pCMV6 (control vector) (4 μg). Forty-eight hours after transfection, HEK293T cells were treated with MG132 (20uM) for 4 h. Cell extracts were immunoprecipitated via incubation with protein G-Sepharose coupled to anti-FLAG antibody; immunocomplexes were separated by SDS-PAGE and analyzed by Western blotting using the anti-FLAG and anti-GSK3β antibodies. (**B**) HEK293T cells (3 × 10^6^) were transfected with pCMV6-IBtkα-FLAG or pCMV6 (4 μg). Forty-eight hours after transfection, HEK293T cells were treated with MG132 (20 µM) or vehicle for 4 h. Cell extracts (30 μg) were separated by Nupage 4–12% polyacrylamide gel followed by Western blotting with antibodies against IBtkα, GSK3β, and GAPDH. (**C**) HEK293T cells (3 × 10^6^) were transfected with HA-tagged ubiquitin and pCMV6-IBtkα-FLAG or pCMV6 (4 μg). Forty-eight hours later, cells were treated with MG132 (20 µM) for 4 h before lysis. Cell extracts were subjected to immunoprecipitation with anti-HA antibody and immunocomplexes were resolved by 6% SDS-PAGE, followed by Western blotting with the antibodies against GSK3β and IBtkα. We use the symbols “+” or “−”to indicate the presence or the absence of MG132, respectively. We use the symbols “+” or “−” to indicate the presence or the absence of each vector, respectively. (**D**) Ramos cells (3 × 10^6^) were transduced with lentiviral particles (500 ng of p24) expressing shCNTL or shIBTK. Twenty-four hours later, transduced cells were selected with puromycin (1.5 μg/mL) and lysed forty-eight hours later. Whole cell extracts (30 µg) of shCNTL or shIBTK Ramos cells were separated by 12% SDS–PAGE and analyzed by Western blotting using anti-IBtkα, anti-GSK3β, and anti-GAPDH antibodies. (**E**) Densitometric values of IBtkα and GSK3β bands were normalized to GAPDH bands. Values (mean ± SE, *n* = 3) are shown. Statistically significant difference was calculated according to Student’s *t* test. (**F**) Total RNA was extracted from shCNTL and shIBTK Ramos cells and analyzed by real-time PCR for the expression of *GSK3β*. Results were normalized using *GAPDH* as the housekeeping gene. Data were statistically analyzed by Student’s *t* test and are reported as mean values ± SE of three independent experiments. Not statistically significant: n.s.

To test this hypothesis, we first verified the physical interaction of IBtkα with GSK3β. HEK293T cells were transfected with the expression vector of IBtkα-FLAG or an empty vector, and were then treated with the 26S proteasome inhibitor MG132. We found that GSK3β was immunoprecipitated with IBtkα-FLAG using anti-FLAG antibody (Figure 2A), confirming our previous MS-based indications (Table S1) [8]. 

Then, we analyzed the GSK3β protein content in HEK293T cells transfected with IBtkα-FLAG or an empty vector, with and without proteasome inhibition. The over-expression of IBtkα leads to the reduction of GSK3β protein in the absence of MG132, whereas the GSK3β levels were restored in the presence of the proteasome inhibitor MG132 (Figure 2B). These results indicate that IBtkα promoted the proteasomal degradation of GSK3β.

Next, we asked whether IBtkα could promote GSK3β ubiquitylation. To this end, we performed ubiquitylation assay in vivo. HEK293T cells were transfected with HA-ubiquitin and IBtkα-FLAG or empty expression vectors, and then treated with MG132. Cell extracts were immunoprecipitated with anti-HA antibody and analyzed by Western blotting with anti-GSK3β antibody. A significant increase of endogenous poly-ubiquitylation forms of GSK3β was observed in cells transfected with IBtkα-FLAG compared to the empty vector (Figure 2C). Then, we verified whether IBtkα could affect the stability of GSK3β protein in cancerous B cells. To this end, Ramos cells were transduced with lentivirus particles expressing either shIBTK or shCNTL and both the GSK3β protein and gene expression levels were analyzed. Using Western blotting analysis, we observed a two-fold increase of GSK3β protein in *IBTK*-silenced cells compared to control cells (Figure 2D). However, the GSK3β mRNA levels were similar in *IBTK*-silenced and unsilenced cells (Figure 2E). Altogether, these results indicate that IBtkα tagged GSK3β to proteasomal degradation by ubiquitylation, without affecting the transcription of the GSK3β gene.

### 2.3. Loss of IBtkα Reduces Β-Catenin Protein and Its Transcriptional Activity

**Figure 3:**
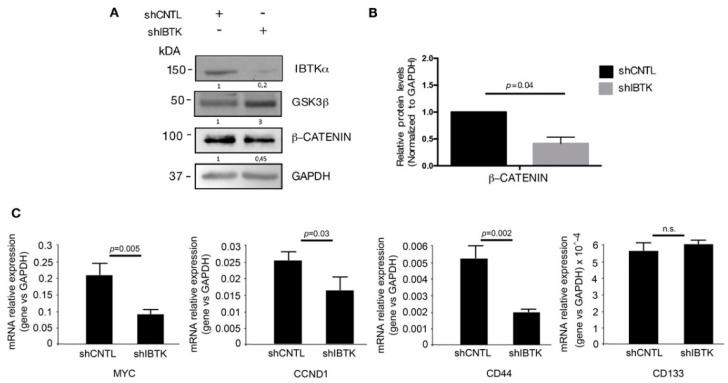
IBtkα silencing decreases the β-catenin protein content and the transcription of β-catenin target genes. Ramos cells (3 × 10^6^) were transduced with lentiviral particles (500 ng of p24) expressing shCNTL or shIBTK. Twenty-four hours later, transduced cells were selected with puromycin (1.5 μg/mL) for 48 h. (**A**) Whole cell extracts (30 µg) were separated by Nupage 4–12% polyacrylamide gel and analyzed using Western blotting with anti-β-catenin, anti-IBTK, anti-GSK3β, and anti-GAPDH antibodies. We use the symbols “+” or “−” to indicate the presence or the absence of each short hairpin RNA, respectively. (**B**) Densitometric values of the β-catenin protein band were normalized to GAPDH bands. Mean values ± SE are shown for three independent experiments. Statistically significant difference was calculated according to Student’s *t* test. (**C**) Total RNA of shCNTL or shIBTK-transduced Ramos cells was analyzed by quantitative real-time PCR for the expression of the β-catenin target genes *MYC*, *CCND1, CD44*, and *CD133*. Values were normalized using GAPDH as the housekeeping gene. Data were statistically analyzed by Student’s *t* test and are reported as mean values ± SE of six independent experiments. Not statistically significant: n.s.

Given that GSK3β is the inhibitor of β-catenin activity, we next tested whether the IBtkα loss could decrease the β-catenin activity by releasing GSK3β. In order to do so, Ramos cells were transduced with lentiviral particles expressing either shIBTK or shCNTL and total cell extracts were analyzed by Western blotting for the levels of IBtkα, GSK3β, and β-catenin. Loss of IBtkα halved the amount of β-catenin protein, while increasing the GSK3β content (Figure 3A,B). These results indicate that IBtkα is required to increase the β-catenin protein levels, likely as a consequence of IBtkα -dependent GSK3β degradation.

In order to provide evidence of a functional contribution the stabilization of β-catenin downstream, we analyzed the effect of IBtkα on the expression of the genes transcriptionally regulated by β-catenin. To this aim, Ramos cells were transduced with shIBTK or shCNTL and total RNAs were analyzed by qRT-PCR for the expression of *MYC*, *CCND1* [16], and *CD44* [22]. The depletion of *IBTK* significantly reduced the expression of the three target genes of β-catenin, without affecting the expression of *CD133*, (Figure 3C). Thus, IBtkα is capable of enhancing the expression of β-catenin target genes as a consequence of GSK3β suppression.

### 2.4. Loss of IBtkα Increases Apoptosis and Reduces Cell Viability

**Figure 4:**
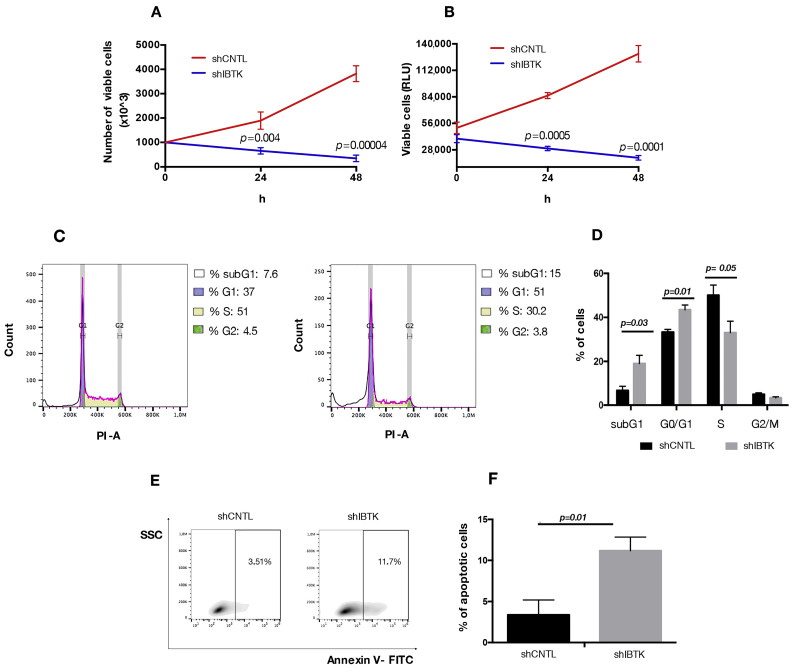
IBtkα silencing decreases cell survival, increasing apoptosis. Ramos cells (3 × 10^6^) were transduced with lentiviral particles (500 ng of p24) expressing shCTRL or shIBTK and twenty-four hours later they were treated with puromycin (1.5 μg/mL) to select stably transduced cells. Cell viability was measured at 0, 24, and 48 h in technical triplicate by trypan blue dye exclusion (**A**) and CellTiter-Glo assay (**B**). Values (mean ± SE; *n* = 3) are shown. Statistically significant difference according Student’s *t* test. (**C**) Representative scatter plot of cell cycle distribution by Propidium Iodide (PI) staining. Briefly, shCNTL or shIBTK-transduced Ramos cells were labeled, fixed, and then stained with PI/RNase staining solution, and cell cycle was analyzed via flow cytometry. The phases of the cell cycle were evaluated by using the Watson pragmatic model within the flow cytometry data analysis software FlowJo Version 10.1. (**D**) A bar diagram of the number of cells in each phase of the cell cycle based on PI staining. Values (mean ± SE; *n* = 3) are shown. Statistically significant difference was according Student’s *t* test. (**E**) Representative density plot of Annexin V binding assay of in vitro cultured shCNTL or shIBTK-transduced Ramos cells analyzed by flow cytometry. (**F**) Bar diagram of the percentage of apoptotic cells (Annexin V positive cells) as measured by Annexin V binding assay. Values (mean ± SE; *n* = 3) are shown. Statistically significant difference was determined by Student’s *t* test.

Our observations relative to the reduced expression of *MYC* and *CCND1* in absence of IBtkα could be relevant for the pro-survival activity of IBtkα, since both *MYC* and *CCND1* are directly involved in cell cycle control in cancerous B cells [23,24]. Thus, we verified the effect of IBtkα depletion on cell viability and division. To this end, Ramos cells were transduced with viral particles expressing shIBTK or shCNTL and analyzed for viability, cell death, and cell cycle. The depletion of IBtkα significantly reduced the number of viable cells, as measured by Trypan blue dye exclusion (Figure 4A). This was confirmed by CellTiter-Glo Luminescent cell viability assay, which is able to infer data about the cohort of metabolically active cells (Figure 4B). In order to further understand whether the slower growth of IBtkα-silenced cells was due to a delay in cell replication or to an increase in cell death, we analyzed the cell cycle. Using flow cytometry, we did observe how the S-phase of cell cycle was significantly reduced in IBtkα-silenced cells compared to the unsilenced control (from 50.1% to 27.2%, *p* value = 0.05), with the concomitant increase of G0/G1population (from 33% to 45.1%, *p* value = 0.01) and subG1 population (from 7.2% to 18.8%, *p* value = 0.03) (Figure 4C). These data indicate that the lack of IBtkα led to a partial block of cell cycle progression from G0 to G1 phase, with the decrease of cells transiting into the S phase. Furthermore, by performing Annexin V binding assay, we observed that the lack of IBtkα increased the percentage of apoptotic rate (11%) of silenced cells compared to unsilenced cells (3%) (Figure 4D,E). Altogether, these results strongly support a direct role of IBtkα leading to a pro-survival effect in cancerous B cells.

## 3. Discussion

*MYC* is a transcription factor that plays a key role in the transcriptional activation of pro-survival and proliferative genes. Regulation of *MYC* activity is essential for the balance of cell life and differentiation. Overexpression of *MYC* occurs in malignant B-cell transformation [25,26]. In particular, duplications and/or translocations of the *MYC* gene are frequently observed in numerous human cancers, such as Burkitt’s lymphoma and multiple myeloma [27,28]. This is consistent with the *MYC*–dependent activation of genes that drive quiescent cells into the cell cycle, thus promoting cell growth [29]. On the other hand, reduced expression levels of *MYC* are associated with non-dividing and differentiated cells [30]. Hence, the possibility of defining further insights into the regulatory mechanism of *MYC* activity would be relevant for B lymphoma malignant transformation.

Expression of the *MYC* gene is under the transcriptional control of β-catenin. β-catenin signaling is upregulated in the progression of many types of cancers, including leukemia [31], myeloma [32,33], and several subtypes of lymphoma [34,35]. In this study, we have addressed the role of IBtkα in the regulation of *MYC* expression. This investigation was prompted by two key observations made by us in some previous studies: firstly, that *Ibtk* synergized with *MYC* in murine B-lymphomagenesis of *Eμ-myc* transgenic mice, and that it was also transcriptionally activated by *MYC* [7,12].

Given that IBtkα is a substrate receptor of Cullin3 ubiquitin ligase [8,36], we addressed the question whether IBtkα could affect the stability of proteins regulating the *MYC* activity. Among putative candidates, we focused on the serine–threonine kinase, GSK3β, which we have previously identified in the characterization of IBtkα interactome via mass spectrometry analysis [8]. As a component of the β-catenin destruction complex, GSK3β phosphorylates β-catenin, tagging it to proteasomal degradation. This event leads to the transcriptional turning-off of genes regulated by β-catenin. Given that *MYC* is among these genes, we were interested in investigating the effect of IBtkα on GSK3β as a repressor of β-catenin activity.

Here, for the first time, we aimed at evaluating the effect of *IBTK* on *MYC* in aggressive lymphoma B cells to elucidate the role of *IBTK* in B-cell lymphoma progression. We expanded our understanding of the mechanistic function of *IBTK* in B-lymphoma, emphasizing its action on the expression of the *MYC* oncogene and determining whether the IBtkα-GSK3β interaction and β-catenin could be involved in this process.

**Figure 5:**
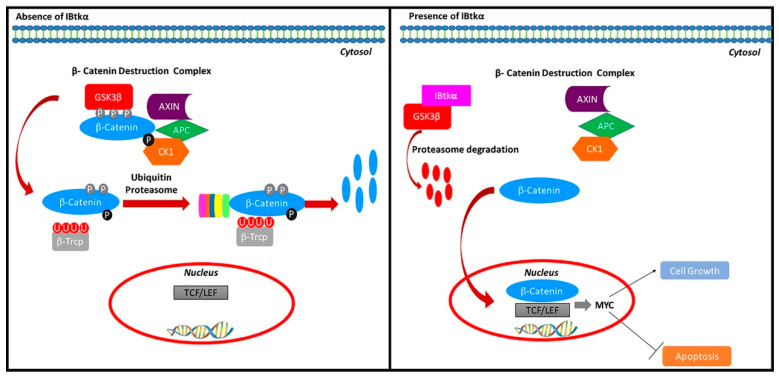
Regulation of the β-catenin pathway by IBtkα. IBtkα associates GSK3β and promotes its ubiquitylation coupled to proteasomal degradation. This event counteracts the GSK3β-dependent phosphorylation of β-catenin within the β-catenin destruction complex. Consequently, β-catenin accumulates in the cytosol, translocates to the nucleus, and associates with Tcf/Lef transcription factors in order to activate the transcription of target genes. Among these genes, *MYC* and *CCND1* promote cell growth and inhibit apoptosis.

With this work, we showed that IBtkα promotes GSK3β degradation, releasing β-catenin from the GSK3β suppression, thus indirectly upregulating *MYC* gene expression (Figure 5). Notably, IBtkα can associate to and promote the ubiquitylation of GSK3β, leading to its proteasomal degradation. In addition, IBtkα silencing increases the GSK3β protein level without modifying the *GSK3β* gene expression. These results indicate that IBtkα exclusively acts at the post-transcriptional level of GSK3β regulation. As a consequence of the involvement of IBtkα in GSK3β degradation, the lack of IBtkα decreases the β-catenin protein level and the expression of the β-catenin target genes *MYC*, *CCND1*, and *CD44*. Furthermore, IBtkα silencing reduces cell viability and increases apoptosis in cancerous B cells. Taken together, our findings demonstrate that the inhibition of IBtkα could have a pro-apoptotic action.

In our previous study, we reported how IBtkα promoted the ubiquitylation and proteasome degradation of Pdcd4, a well-known inhibitor of the eIF4A1 helicase [8], and consequently, it released the translation of transcripts under the inhibitory control of Pdcd4, such as the anti-apoptotic Bcl-XL protein [8]. Here, we have shown that IBtkα promotes the ubiquitylation and proteasome degradation of the β-catenin inhibitor GSK3β and consequently activates the β-catenin-dependent gene expression of *MYC*, *CCND1*, and *CD44* genes. Altogether, our findings highlight IBtkα as a master regulator of both transcription- and translation-dependent cellular functions, which are relevant for B-cell growth control. Thus, it is tempting to speculate how drugs capable of reducing IBtkα expression levels could represent encouraging chemotherapeutic agents in the clinical treatment of B-cell lymphoma.

Nonetheless, attention should be paid to the use of *IBTK* as a therapeutic agent as there might be multiple adverse effects. This may be due to the fact that GSK3β is intimately related to several signaling pathways, including, among others, the PI3K/AKT/mTOR [37] and the NF-kB modules [38]. GSK3β plays a role in several different biochemical processes, such as protein [39] and lipid synthesis [40], glucose [41] and mitochondrial metabolism [42], and autophagy [43]. In this view, the IBtkα-dependent degradation of GSK3β could affect different cellular pathways, and so, we believe, significantly expand our understanding of the molecular mechanisms underlying its pro-survival activity.

## 4. Materials and Methods

### 4.1. Cells, Plasmids, Lentivirus, Antibodies

HEK293T, Ramos, and Raji cells were purchased from Sigma-Aldrich. Ramos and Raji cells were grown in RPMI (Thermo Fisher Scientific, Waltham, MA, USA). HEK293T cells were grown in Dulbecco’s Modified Eagle Medium (DMEM; Thermo Fisher Scientific, Waltham, MA, USA). Cell culture media were supplemented with 10% fetal bovine serum (FBS), 2 mM L-glutamine, 1 mM Na-pyruvate, 50 mM 2−mercaptoethanol, 100 U/mL penicillin, and 100 μg/mL streptomycin; all reagents were purchased from Thermo Fisher Scientific. The plasmids pCMV6-IBtkα-FLAG and pCMV6 were from OriGene Technologies, Inc. (Rockville, MD, USA). The lentiviral constructs expressing the short hairpin RNA against IBtkα (shIBTK) or control short hairpin RNA (shCNTL) (TRCN0000082575 and SHC002, respectively) were from MISSION^®^ (Sigma-Aldrich, St. Louis, MO, USA).

### 4.2. Cells Transfection, Transduction, and Treatments

HEK293T cells were transfected with plasmids using Lipofectamine 2000 (Thermo Fisher Scientific), according to the manufacturer’s protocol. Lentiviral particles were produced by transfection of HEK293Tcells, as previously described [44,45]. Briefly, HEK293Tcells (1 × 10^6^) were transfected with pCMV-dR8.91 (5 μg) and pCMV-VSVG (5 μg) together with shIBTK (10 μg) or shCNTL (10 μg); 48 h post-transfection, cell supernatant was collected, filtered through 0.22 μ sterile filter, and used for spinoculation in the presence of 8 μg/mL polybrene. For IBtkα silencing, Ramos and Raji cells (3 × 10^6^) were transduced with lentiviral particles (500 ng of p24) expressing shIBTK or shCNTL. Twenty-four hours later, transduced cells were subjected to selection with puromycin (1.5 μg/mL and 0.2 μg/mL, respectively) for 48 h. When required, cells were treated with the proteasome inhibitor MG132 (Sigma-Aldrich). The RNA transcription inhibitor actinomycin D was from Abcam (Cambridge, UK).

### 4.3. Mice and Isolation of B Lymphoma Cells

*Ibtk^+^*^/*+*^*Eμ-myc* and *Ibtk^−^*^/*−*^*Eμ-myc mice* were previously described [7]. Murine B cells were isolated from tumor lymph nodes of *Ibtk^+^*^/*+*^*Eμ-myc* and *Ibtk^−^*^/*−*^*Eμ-myc* mice through depletion of non-B cells using magnetic-activated cell sorting (MACS) B-cell isolation kit and MS columns (Miltenyi Biotech, Bergisch Gladbach, Germany), according to the manufacturer’s protocols. Flow cytometry from MACS separated cells revealed 95% purity of B cells.

### 4.4. Quantitative Real-Time PCR

Quantitative real-time PCR (qRT-PCR) was performed as previously described [46]. Briefly, total RNA was isolated from cells using GenElute Mammalian Total RNA Miniprep reagent (Sigma-Aldrich). After DNase treatment, cDNA was synthesized by the RT^2^ First Strand kit (SABiosciences, MD, USA), according to the manufacturer’s instructions. Real-time PCR was performed with the PowerUP Sybr green master mix (Thermo Fisher Scientific) using a Quant Studio 7 Flex instrument and fast gene expression method: 95 °C, 20′′; (95 °C, 1′′; 60 °C, 20′′) × 40 cycles; 95 °C, 15′′; 60 °C 1′; 0.05 °C/s up to 95 °C. Real-time PCR results were analyzed using Quant Studio Real-Time PCR Software (Thermo Fisher Scientific). Reactions were carried out in triplicate and gene expression levels were calculated relatively to *GAPDH* mRNA levels as endogenous control. Real-time PCR amplification values were reported as 2^−ΔCt^, where Δ_Ct_ is Ct_gene_ under investigation of −Ct_endogenous_ control [47]. The following primers were used: human *MYC* gene, 5′-AAAGGCCCCCAAGGTAGTTA-3′ and 3′-GCACAAGAGTTCCGTAGCTG-5′ (153 bp products) [48]; human *GSKβ* gene, 5′-GGAACTCCAACAAGGGAGCA-3′ and 3′-TTCGGGGTCGGAAGACCTTA-5′ (106 bp products); human *CCND1* gene, 5′-GGTGTCCTACTTCAAATGTGTGC-3′ and 3′-GTCTCCTTCATCTTAGAGGCCAC-5′; human *CD44* gene 5′-GTGATGGCACCCGCTATGTC-3′ and 3′-AACCTCCTGAAGTGCTGCTCC-5′; human *CD133* gene 5′-TCAGGATTTTGCTGCTTGTG-3′ and 3′-GCAGTATCTAGAGCGGTGGC -5′ [49].

### 4.5. Immunoprecipitation and Western Blotting

Cells were lysed in modified RIPA buffer (10 mM Tris-HCl, pH 7.5, 150 mM NaCl, 1 mM EDTA, 1% Igepal). Immunoprecipitation (IP) experiments were performed as previously described [50,51]. Briefly, cells were lysed in RIPA buffer (50 mM Tris-HCl, pH 8.0, 150 mM NaCl, 1 mM EDTA, 1% Igepal, 0.5% sodium deoxycholate). Protein extraction was performed in the presence of a protease inhibitor cocktail (Roche Diagnostic GmbH, Mannheim, Germany) and 2 mM N-Ethylmaleimide (Sigma-Aldrich), using 1 mL of cold buffer for 100 mm dish. Cell lysates were clarified by centrifugation at 14,000× *g* for 10 min, and overnight incubated with the appropriate antibody, followed by a two-hour incubation with G-protein beads (30 μL sample) (GE Healthcare, Buckinghamshire, UK). The beads were washed 5 times with 1 mL of cold RIPA buffer, and denatured for 10 min at 70 °C in 25 μL of 2× Nupage sample buffer (Life Technologies). Protein samples were subjected to electrophoresis on Nupage 4–12% polyacrylamide gel (Life Technologies) or self-casted 6% polyacrylamide gel, and then transferred onto a nitrocellulose membrane (GE Healthcare). Antibodies were: anti-IBtk (#A303-001A; Bethyl Laboratories, Inc., Montgomery, TX, USA), anti-Myc (#5605; Cell Signaling Technology), anti-GSK3β (#9315; Cell Signaling Technology), anti-GAPDH (#sc-47724; Santa-Cruz Biotechnology, Dallas, TX, USA), anti-vinculin (V9131, Sigma-Aldrich).

### 4.6. Cell Viability, Apoptosis and Cell Cycle Analysis

Cell viability was determined using trypan blue dye exclusion and CellTiter-Glo^®^ Luminescent Cell Viability Assay (Promega, Madison, WI, USA), which is based on quantitation of ATP as an indicator of metabolically active cells. Annexin V-based apoptotic assay was performed as previously described [52]. Briefly, Ramos cells (1 × 10^6^) were stained with FITC-conjugated Annexin V and propidium iodide (PI) using the Annexin V-FITC kit (Miltenyi Biotech). Data were collected by flow cytometry. Cell cycle analysis was performed as previously described [7,53]. Briefly, cells were fixed with 70% (*v*/*v*) cold ethanol and stored at −20 °C for 1 h. Then, cells were washed with cold PBS, centrifuged and the pellets were suspended in 200 μL of non-lysis solution containing PI (50 μg/mL) and RNase (250 μg/mL.) After incubation at 4 °C for 30 min, cells were analyzed by flow cytometer (BriCyteE6).

### 4.7. Statistical Analysis

Statistical analysis was performed by the two-tailed unpaired Student’s *t* test using GraphPad Prism^®^ software package. Statistical significance was determined by *p* < 0.05.

## Data Availability

The data presented in this study are available on request from the corresponding author.

## Supplementary Materials

The following supporting information can be downloaded at: www.mdpi.com/article/10.3390/ijms23042044/s1.
