# Supplementary material for: IBtkα Activates the β-Catenin-Dependent Transcription of MYC through Ubiquitylation and Proteasomal Degradation of GSK3β in Cancerous B Cells
Source: Int J Mol Sci. 2022 Feb 12;23(4):2044. doi: 10.3390/ijms23042044 (PMC8875111; doi:10.3390/ijms23042044)
Supplement: Supplementary file 1 [file ijms-23-02044-s001.zip › ijms-1571511-supplementary.pdf]

International Journal of *Molecular Sciences*

MDPI

Supplementary materials

IBtkα Activates the β–Catenin-Dependent Transcription of MYC through Ubiquitylation and Proteasomal Degradation of GSK3β in Cancerous B Cells

Eleonora Vecchio [1,*], Nancy Nisticò [1], Gaetanina Golino [1], Enrico Iaccino [1], Domenico Maisano [1], Selena Mimmi [1], Annamaria Aloisio [1], Maurizio Renna [2], Angelica Avagliano [3], Alessandro Arcucci [3], Giuseppe Fiume [1,*,†] and Ileana Quinto [1,†]

[1] Department of Experimental and Clinical Medicine, University of Catanzaro 'Magna Graecia', 88100 Catanzaro, Italy nancynistico@unicz.it (N.N.); tania.golino@gmail.com (G.G.); iaccino@unicz.it (E.I.); maisano@unicz.it (D.M.); mimmi@unicz.it (S.M.); aloisio@unicz.it (A.A.); quinto@unicz.it (I.Q.)
[2] Department of Molecular Medicine and Medical Biotechnology, University of Naples Federico II, 80131 Naples, Italy; maurizio.renna@unina.it (M.R.)
[3] Department of Public Health, University of Naples Federico II, 80131 Naples, Italy; angelica.avagliano@unina.it (A.A.); alessandro.arcucci2@unina.it (A.A.)
* Correspondence: eleonoravecchio@unicz.it (E.V.); fiume@unicz.it (G.F.)
† G.F. and I.Q. should be considered as co-last authors.

Table S1. Mass spectrometry-based identification of GSK3β from the IBtkα co-immunoprecipitation experiment. HEK293T cells were transfected with pCMV6-IBtkα-FLAG plasmid or the corresponding empty vector (24 μg of DNA/100-mm dish). Protein extracts (1,5 mg) were immunoprecipitated with anti-FLAG antibody (20 μg), and the immunocomplexes were resolved by NuPAGE 4–12% SDS-PAGE. Gels were stained with colloidal Coomassie, and protein bands were excised for in-gel tryptic digestion. Chromatography of tryptic peptides and mass spectrometry were performed as previously described [8].

Accession no.	Description	Coverage	Unique Peptides
Q9P2D0	Inhibitor of Bruton tyrosine kinase	60.16	110
P49841	Glycogen synthase kinase-3 beta	7.88	2
